# Design and Control of a New Biomimetic Transfemoral Knee Prosthesis Using an Echo-Control Scheme

**DOI:** 10.1155/2018/8783642

**Published:** 2018-04-30

**Authors:** Mario G. Bernal-Torres, Hugo I. Medellín-Castillo, Juan C. Arellano-González

**Affiliations:** Facultad de Ingeniería, Av. Dr. Manuel Nava No. 8, Universidad Autónoma de San Luis Potosí, 78290 San Luis Potosí, SLP, Mexico

## Abstract

Passive knee prostheses require a significant amount of additional metabolic energy to carry out a gait cycle, therefore affecting the natural human walk performance. Current active knee prostheses are still limited because they do not reply with accuracy of the natural human knee movement, and the time response is relatively large. This paper presents the design and control of a new biomimetic-controlled transfemoral knee prosthesis based on a polycentric-type mechanism. The aim was to develop a knee prosthesis able to provide additional power and to mimic with accuracy of the natural human knee movement using a stable control strategy. The design of the knee mechanism was obtained from the body-guidance kinematics synthesis based on real human walking patterns obtained from computer vision and 3D reconstruction. A biomechanical evaluation of the synthesized prosthesis was then carried out. For the activation and control of the prosthesis, an echo-control strategy was proposed and developed. In this echo-control strategy, the sound side leg is sensed and synchronized with the activation of the knee prosthesis. An experimental prototype was built and evaluated in a test rig. The results revealed that the prosthetic knee is able to mimic the biomechanics of the human knee.

## 1. Introduction

Advanced design and manufacture engineering techniques, such as computer-aided design (CAD), computer-aided engineering (CAE), image processing and 3D reconstruction, reverse engineering (RE), robotics and computer vision, virtual and augmented realities (VR and AR, resp.), and additive manufacturing (AM), have been created and developed to assist industry. However, the use of these modern engineering technologies in biomedical applications for the delivery of human healthcare has increased exponentially in the last three decades. As a consequence, the engineering-assisted surgery (EAS) was introduced as a new research field and defined as the application of engineering and manufacturing technology in the delivery of healthcare [1]. EAS comprises of data acquisition from CT, MRI, and scanners; rapid prototyping and manufacturing (RP&M); CAD; reverse engineering (RE); and finite element analysis (FEA). The aim is to enhance the healthcare conventional processes. Typical applications of engineering technologies in medicine include visualization and reconstruction of human anatomy; organ and tissue modeling; surgical simulators for planning and training; robotic surgery; locomotion and gait analysis; and design and manufacture of prostheses, ortheses, implants, biomodels, scaffolds, training models, and surgical aids and tools.

In the area of prostheses and implants, traditional fabrication methods are expensive in terms of time and cost, and the accuracy of the prosthesis or implant is limited to the experience and manual sculpting skills of the specialist [2]. Moreover, poorly shaped prostheses and implants are difficult to locate and secure in place, increasing the surgical time and risk of infection, and in some cases, they are also ineffective [3]. Thus, in order to enhance the quality and effectiveness of prostheses and implants, several research works have focused on the development of new approaches for the design and fabrication of prostheses and implants based on the use and integration of engineering technologies, for example [2–15]. The results have shown that modern engineering technologies allow the design and fabrication of customized prostheses and implants, with benefits in terms of effectiveness, accuracy, fabrication and implantation time, cost, and safety.

On the other hand, knee prostheses are one of the most extensively used prosthesis devices. They are divided into two types: external and internal. Internal knee prostheses are used to repair a damaged human knee, while external knee prostheses are vital for knee amputees in order to recover their walking ability. According to the type of mechanism and mobility, external knee prostheses can be further subdivided into two types: single-axis prostheses, also known as hinge-type, and polycentric-type prostheses (or more than one axis) [16]. Single-axis knee prostheses work as a hinge, allowing flexion and extension by the rotation around one axis. On the other hand, polycentric-type knee prostheses are more complex but allow greater accuracy than single-axis knee prostheses; the control of the centrode's shape; a good stability during the first part of the stance phase (at the beginning of the step); and a reduction of the leg length at the start of the stride, decreasing the risk of stumbling (leg-shortening effect) [17, 18]. Thus, it is evident that polycentric-type knee prostheses offer superior capabilities than the single-axis prostheses; however, its design, control, and manufacture are more complex and costly.

Knee prostheses can also be classified based on the energy usage as passive, dynamically damped, and active prostheses. Passive and dynamically damped knees are not able to provide external power during the gait cycle, and therefore, they cannot replicate the generative phases of the natural knee [19]. Most of the current commercial transfemoral prostheses remain limited to energetically passive devices in which the joints can store and dissipate energy but cannot provide extra power. This inability to provide extra energy significantly impairs the capability of the prosthesis to develop some locomotive functions, including stair and ramp climbing. Moreover, a person who uses a passive prosthesis with cadence and friction control spends up to 60% more metabolic energy to complete a gait cycle than healthy subjects [20]. Another disadvantage of nonpowered knee prostheses is the insufficient symmetry of the gait, which, in the long term, will affect the nonimpaired leg.

An active prosthesis with the capacity to deliver power to the knee and ankle joints would presumably reduce the deficiencies of passive prostheses and would additionally enable the restoration of biomechanically normal locomotion [20–24]. An investigation of the gait symmetry revealed that microprocessor-controlled prosthetic knees have superior performance and more biomechanical advantages than passive knee prostheses [21]. These advantages include smoother gait, less compensator hip activity on the contralateral side, and the reduction of the stumbling and fall risk [25, 26]. Therefore, powered knee prostheses are becoming more popular, but they comprise several engineering challenges, mainly due to energy, control, and weight issues. However, recent developments in actuators, sensors, and energy storage technologies could alleviate the solution of these issues.

This paper presents the design and analysis of a new biomimetic-controlled transfemoral knee prosthesis. The aim was to develop a new knee prosthesis able to provide additional power and to mimic with accuracy the natural human knee movement. The prosthesis development is based on the use and integration of computer vision, image processing, CAD, CAE, and mechatronics technologies. The knee mechanism is a polycentric planar linkage obtained from the kinematic and synthesis analysis using computer vision and 3D reconstruction of human walking patterns. A biomechanical evaluation of the synthesized prosthesis is also presented in order to compare its performance with the performance of a natural knee.

## 2. Related Works

In order to restore the gait function after a transfemoral amputation, several active exoprostheses able to provide and control the knee torque have been developed. At the moment, numerous strategies to control active transfemoral prosthesis have been proposed, and some of them include electromyography (EMG) signals, finite-state controllers, echo control, trajectory control, and complementary limb motion estimation (CMLE), among others. An EMG position control system for an active ankle-foot prosthetic was presented in [27]. However, one major disadvantage of using an EMG-based control system is the high noise sensitivity, especially when noninvasive methods are used. Furthermore, the accuracy of the EMG signal identification and characterization corresponding to gait patterns is still unsatisfactory. As a result, the response time of the prosthesis is sometimes not instantaneous, causing frustrated movements and instability. On the other hand, a trajectory-based control scheme for a knee joint prosthesis was presented in [28]. In this study, the application of predefined patterns of lower limb activities to control the active above-knee prosthesis (AKP) was implemented. Several scripts related to the basic daily living activities of the amputee were generated. However, these predefined patterns imply a major disadvantage because they limit the intention and freedom of the patient, restricting the natural biomimetization of human gait. According to [29], the rule-based phase detection algorithms are based on the recognition of peaks in the logged signals, which complicates its use in real-time operation.

A finite-state approach to control an active prosthesis during walking and standing was reported in [20, 30, 31]. This control strategy utilizes an impedance-based approach to generate the joint torques for each activity mode (walking, running, stair climbing, etc.). These torques are governed by separated finite-state machines, which modulate the joint impedance according to the gait phase. Thus, the impedance control approach is able to provide a functional gait that is representative of the normal gait biomechanics. However, the kinematic behavior of the prostheses used in these works did not biomimic the natural leg-shortening effect during walking.

A prosthesis with an agonist-antagonist knee to restore the knee function via biomimetic actuation was reported in [32]. The actuation of the agonist-antagonist AKP comprises of a pair of elastic actuators, allowing the use of a quasi-passive control scheme on level ground walking, which is achieved by the use of a finite-state machine. Nevertheless, the knee prosthesis used in this investigation did not mimic the natural leg-shortening effect. Based on the interjoint coordination in physiological gait, a statistical regression to estimate the missing motion was used in [33]. By means of a mapping function, the residual body motion was mapped to the prosthesis, and the amputee patient was able to achieve a nearly physiological gait pattern. Furthermore, in a pilot study, this algorithm, called complementary limb motion estimation (CMLE), was applied to control an active knee exoprosthesis.

An investigation to evaluate the feasibility of using an echo-control strategy in a prosthetic knee was presented in [34]. In this study, the sensory system specifications and the requirements to measure the limb posture were investigated. Four combinations of sensors (digital protractor, a combination of two accelerometers, potentiometer, and potentiometer plus accelerometer) were analyzed and tested in order to find the best configuration for signal acquisition and robust control. The results showed that the most suitable way to detect the leg posture was the combination of an accelerometer with another sensor (diversity principle), in this case, a potentiometer. The commercially available Power Knee™ by Össur® uses an echo-type control approach based on the sensing of the sound side leg. Depending on the phase of the gait cycle and the posture of the limb, the microprocessor determines the required position of the knee and sends this information to the actuator, which moves the joint to the defined position. As most of the microprocessor knees, the major disadvantage of this prosthesis is the high cost and the limited motion of the hinge-type mechanism.

From this literature review, the advantages of active knee prostheses including the enhancement of passive prostheses can be remarked. It is also noticed that most of the existing powered knee prostheses proposed in the literature are based on a hinge-type mechanism due to the simplicity of the control. However, this hinge-type prosthesis does not biomimic the natural centroid shape and leg-shortening effect during the swing phase, increasing the risk of stumbles and falls. It is also observed that the use of predefined leg trajectories could cause asymmetry during gait, inducing long-term injuries in the nonimpaired limb. Therefore, there is still the need to develop active knee prostheses with stable control strategies and capable of efficiently detecting human gait. It is believed that the development of such prostheses would enhance the rehabilitation process and daily living performance of amputees. To overcome these limitations, the authors have proposed a new concept based on the combination of a polycentric knee prosthesis and an echo-control strategy [35].

## 3. Prosthesis Design

### 3.1. Human Gait Analysis

The knee joint is one of the critical elements in the design of transfemoral prostheses because it is responsible for the stability and mobility of the leg during the gait cycle [36]. There are several kinematical criteria to design a polycentric knee mechanism. Some of these criteria include the orientation and position of the leg, the leg-shortening effect, the centrode shape, and the position of the ICR (instantaneous centre of rotation). From a kinematical point of view, it could be possible that not all criteria are fulfilled and globally optimal. Research results on the characterization and analysis of human walking patterns have revealed that one of the most crucial factors is the leg-shortening effect [16, 37].

In a preliminary research work conducted by the authors [16], real human gait patterns were reconstructed and analyzed using a marker-based 3D computer vision system. It was observed that the largest leg length occurred at the stance phase while the shortest length occurred during the knee flexion movement; that is, the knee joint reduces the leg length during the swing phase to prevent stumbles and falls. Another important observation was the maximum deviation between the tibia and femur, detecting an average of 5.3° between the sagittal plane and the tibia. This angle suggests a second degree of freedom around the frontal axis. Since the prosthetic knee should mimic the natural biomechanics of the knee, including the centrode shape and the leg-shortening effect, a polycentric-type mechanism is proposed because of its superior kinematic capabilities than a hinge-type mechanism [18, 36, 38].

### 3.2. Kinematic Synthesis

**Figure 1:**
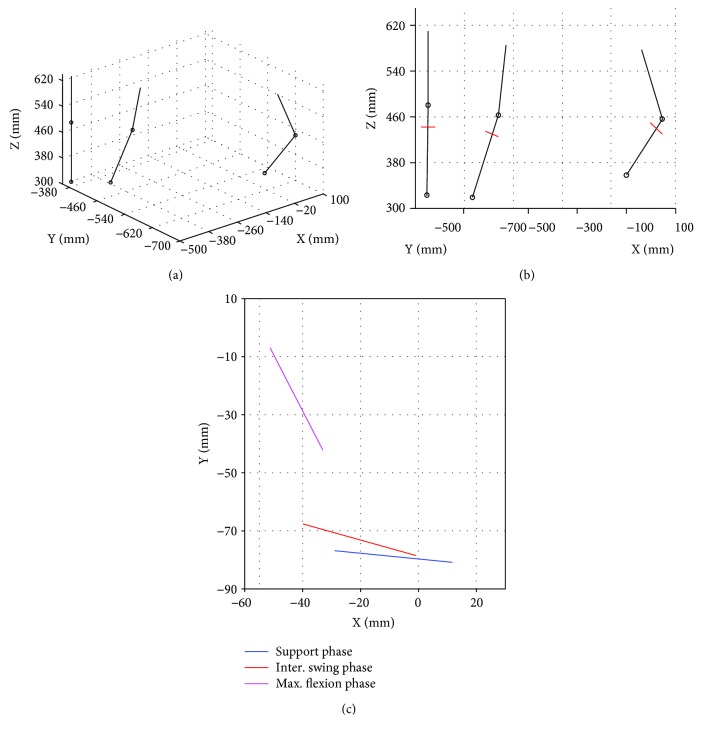
Desired poses: (a) 3D view, (b) sagittal plane view with the addition of the coupler link, and (c) desired poses of the coupler link.

In order to determine the dimensions of the prosthetic mechanism, a body-guidance dimensional synthesis problem for a four-bar linkage is formulated. For this purpose, the driven body is defined as the tibia-ankle-foot prosthetic member, which must be moved through all the desired configurations. Three desired configurations or poses are defined based on the results presented in [16]. The three desired poses are shown in Figure 1(a). A straight line perpendicular to the tibia and with a length of 40 mm was placed in each pose, as shown in Figure 1(b). These perpendicular lines represent the coupler link poses that the synthesized mechanism shall satisfy, as shown in Figure 1(c). These selected poses correspond to the critical phases of a human gait: the stance phase, the transition phase (between phase support and swing), and the average maximum swing phase; this latter phase is reached when the amplitude of the average flexion motion between the femur and tibia is maximum.

**Figure 2:**
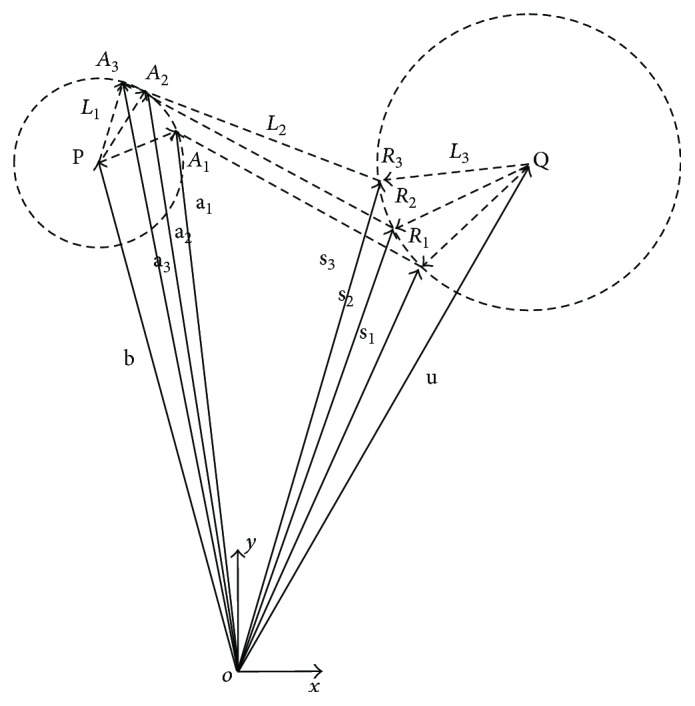
Three desired positions of the proposed four-bar mechanism.

To carry out the body-guidance dimensional synthesis, the diagram shown in Figure 2 is used. The input link is defined as *L*_1_, the coupler link as *L*_2_, the output link as *L*_3_, and the fixed link as *L*_4_ (not shown); O represents the origin of the *X*-*Y* coordinate system; P is the fixed joint of link *L*_1_; **b** is the vector that defines the position of joint P; A_1_, A_2_, and A_3_ are the desired (known) positions of the mobile joint A between *L*_2_ and *L*_1_; **a**_1_, **a**_2_, and **a**_3_ are the vectors that define the three locations of joint A; Q is the fixed joint of *L*_3_; **u** is the vector that defines the position of point Q; R_1_, R_2_, and R_3_ are the desired configurations of joint R between *L*_3_ and *L*_2_; and **s**_1_, **s**_2_, and **s**_3_ are the vectors that define the locations of joint R.

From Figure 1(c), the desired configurations of the coupler link are obtained together with the location of joints A and R at the desired poses, A_*i*_ and R_*i*_, as shown in Table 1. Thus, the synthesis problem is formulated as follows: given the positions and orientations of the coupler link *L*_2_ (Table 1), determine the location of the fixed joints P and Q and the link lengths of the mechanism.

To solve the synthesis problem, it is assumed that the length of the input link is constant. Thus, the following equations are derived from Figure 2:

(1) $$\begin{array}{c} \left(a_{1}-b\right)\cdot \left(a_{1}-b\right)=\left(a_{2}-b\right)\cdot \left(a_{2}-b\right),\\ \left(a_{1}-b\right)\cdot \left(a_{1}-b\right)=\left(a_{3}-b\right)\cdot \left(a_{3}-b\right). \end{array}$$

These equations represent a set of two equations with two unknowns, **b**_*x*_ and **b**_*y*_, which are the components of vector **b** that define the location of the fixed joint P. Once **b** is solved, the length of the input link can be computed as

(2) $$\begin{array}{c} L_{1}=\sqrt{{\left(a_{ix}-b_{x}\right)}^{2}+{\left(a_{iy}-b_{y}\right)}^{2}}, \end{array}$$

where **a**_*ix*_ and **a**_*iy*_ are the *x* and *y* components of vector **a**_*i*_. Similarly, to find the location of joint Q, it is assumed that the output link has a constant length. Thus, the following equations are derived:

(3) $$\begin{array}{c} \left(s_{1}-u\right)\cdot \left(s_{1}-u\right)=\left(s_{2}-u\right)\cdot \left(s_{2}-u\right),\\ \left(s_{1}-u\right)\cdot \left(s_{1}-u\right)=\left(s_{3}-u\right)\cdot \left(s_{3}-u\right). \end{array}$$

The components of vector **u** (**u**_*x*_ and **u**_*y*_), which define the location of the fixed joint Q, can be obtained by solving these two equations. Thus, the length of link *L*_3_ can be estimated as

(4) $$\begin{array}{c} L_{3}=\sqrt{{\left(s_{ix}-u_{x}\right)}^{2}+{\left(s_{iy}-u_{y}\right)}^{2},} \end{array}$$

where **s**_*ix*_ and **s**_*iy*_ are the *x* and *y* components of vector **s**_*i*_. Finally, the length of the fixed link *L*_4_ is calculated as the distance between the joints P and Q:

(5) $$\begin{array}{c} L_{4}=\sqrt{{\left(b_{x}-u_{x}\right)}^{2}+{\left(b_{y}-u_{y}\right)}^{2}}. \end{array}$$

**Figure 3:**
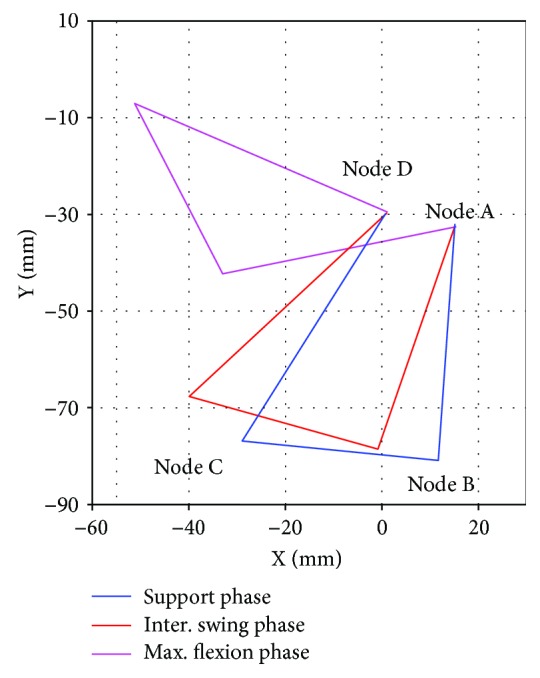
Gait phases of the synthesized mechanism.

The results of the kinematic synthesis problem are summarized in Table 2. These results were obtained assuming a 40 mm length coupler link. To validate this solution, the numerical simulation of the synthesized mechanism was carried out, and the results are presented in Figure 3. From these results, it is observed that the synthesized mechanism is able to achieve the three desired gait configurations.

### 3.3. Kinematic Analysis

**Figure 4:**
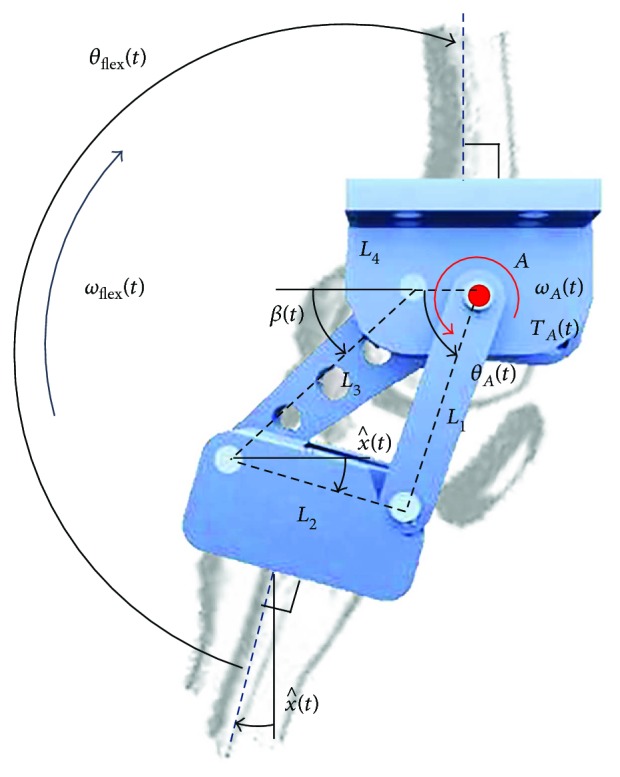
Kinematic diagram of the proposed knee mechanism.

A kinematic analysis of the synthesized four-bar mechanism was carried out to calculate the angular position and the angular velocity at input joint A, *θ*_A_(*t*) and *ω*_A_(*t*), respectively, as a function of the knee flexion-extension angle *θ*_flex_(*t*) and angular velocity *ω*_flex_(*t*) during gait, see Figure 4. From this figure, it is observed that

(6) $$\begin{array}{c} \theta_{flex}\left(t\right)=\pi -\hat{x}\left(t\right), \end{array}$$

where $\hat{x}\left(t\right)$ denotes the tibia tilt angle. By deriving (6) with respect to time, the following expression is obtained.

(7) $$\begin{array}{c} \omega_{flex}\left(t\right)=-\dot{\hat{x}}\left(t\right), \end{array}$$

which means that *θ*_flex_(*t*) and *ω*_flex_(*t*) can be expressed in terms of $\hat{x}\left(t\right)$ and $\dot{\hat{x}}\left(t\right)$.

The following two nonlinear constraint equations are obtained from Figure 4.

(8) $$\begin{array}{c} L_{1}\cos\theta_{A}+L_{2}\cos\hat{x}-L_{3}\cos\beta -L_{4}=0,\\ L_{1}\sin\theta_{A}-L_{2}\sin\hat{x}-L_{3}\sin\beta =0. \end{array}$$

By solving (8), an expression for *θ*_A_(*t*) in terms of $\hat{x}\left(t\right)$ is obtained.

(9) $$\begin{array}{c} \theta_{A}\left(t\right)=2\arctan\left(\frac{-A\pm \sqrt{A^{2}+B^{2}-C^{2}}}{C-B}\right), \end{array}$$

where

(10) $$\begin{array}{c} A=-2L_{1}L_{2}\sin\hat{x},\\ B=2L_{1}L_{2}\cos\hat{x}-2L_{1}L_{4},\\ C={L_{1}}^{2}+{L_{2}}^{2}+{L_{4}}^{2}+{L_{3}}^{2}-2L_{2}L_{4}\cos\hat{x}. \end{array}$$

On the other hand, solving for *β*(*t*) is as follows.

(11) $$\begin{array}{c} \beta \left(t\right)=2\arctan\left(\frac{L_{1}\sin\theta_{A}-L_{2}\sin\hat{x}}{L_{3}+L_{1}\cos\theta_{A}+L_{2}\cos\hat{x}-L_{4}}\right). \end{array}$$

Deriving (8) with respect to time and considering that $\dot{\hat{x}}\left(t\right)$ is given by (7), the next system of equations is obtained.

(12) $$\begin{array}{c} \left[\begin{array}{cc} -L_{1}\sin\theta_{A} & L_{3}\sin\beta \\ L_{1}\cos\theta_{A} & L_{3}\cos\beta \end{array}\right]\left\{\begin{array}{c} {\dot{\theta }}_{A}=\omega_{A}\\ \dot{\beta } \end{array}\right\}=\left\{\begin{array}{c} L_{2}\dot{\hat{x}}\sin\hat{x}\\ L_{2}\dot{\hat{x}}\cos\hat{x} \end{array}\right\}. \end{array}$$

Similarly, deriving this equation system with respect to time and considering that $\ddot{\hat{x}}\left(t\right)$ is obtained from deriving (7), the expressions for the angular acceleration are found as follows.

(13) $$\begin{array}{c} \left[\begin{array}{cc} -L_{1}\sin\theta_{A} & L_{3}\sin\beta \\ L_{1}\cos\theta_{A} & L_{3}\cos\beta \end{array}\right]\left\{\begin{array}{c} {\ddot{\theta }}_{A}=\alpha_{A}\\ \ddot{\beta } \end{array}\right\}=\left\{\begin{array}{c} L_{1}{\dot{\theta }}_{A}^{2}\cos\theta_{A}+L_{2}\ddot{\hat{x}}\sin\hat{x}+L_{2}{\dot{\hat{x}}}^{2}\cos\hat{x}-L_{3}{\dot{\beta }}^{2}\cos\beta \\ L_{1}{\dot{\theta }}_{A}^{2}\sin\theta_{A}+L_{2}\ddot{\hat{x}}\cos\hat{x}-L_{2}{\dot{\hat{x}}}^{2}\sin\hat{x}-L_{3}{\dot{\beta }}^{2}\sin\beta \end{array}\right\}. \end{array}$$

Thus, the input movements of the prosthesis mechanism *θ*_A_(*t*), *ω*_A_(*t*), and *α*_A_(*t*), needed to satisfy a knee movement, *θ*_flex_(*t*) or *ω*_flex_(*t*), can be calculated using (6), (7), (9), (12), and (13).

### 3.4. Biomechanical Evaluation

#### 3.4.1. Flexion-Extension Motion

The kinematic characterization of the natural knee flexion-extension motion was reported in [28], where the angle between the femur and tibia on the sagittal plane *θ*_flex_(*t*), during a complete gait cycle and with an average cadence of one-step per second, was reported. This flexion-extension motion is approximated using a nonlinear curve fitting process and five intervals of 0.2 s each. As a result, the following two sets of five equations (one equation per interval) are obtained for the knee angular position and velocity *θ*_flex_(*t*) and *ω*_flex_(*t*).

(14) $$\begin{array}{c} \theta_{flex}\left(t\right)=\left\{\begin{array}{c} 3\times 10^{3}t^{3}-955t^{2}+1.4\times 10^{-14}t+180\\ 3.1\times 10^{3}{\left(t-0.2\right)}^{3}+866{\left(t-0.2\right)}^{2}-19t+177\\ 2\times 10^{3}{\left(t-0.4\right)}^{3}-988{\left(t-0.4\right)}^{2}-44t+199\\ 2.6\times 10^{3}{\left(t-0.6\right)}^{3}+222{\left(t-0.6\right)}^{2}-200t+266\\ -7.5\times 10^{3}{\left(t-0.8\right)}^{3}+1.8\times 10^{3}{\left(t-0.8\right)}^{2}+200t-29 \end{array}\right\}, \end{array}$$

(15) $$\begin{array}{c} \omega_{flex}\left(t\right)=\left\{\begin{array}{c} 333t^{3}-144t^{2}+1.2t+1.8\\ 199{\left(t-0.2\right)}^{3}+59{\left(t-0.2\right)}^{2}-15t+2.1\\ -1.1\times 10^{3}{\left(t-0.4\right)}^{3}+177{\left(t-0.4\right)}^{2}+32t-13\\ 1.7\times 10^{3}{\left(t-0.6\right)}^{3}-466{\left(t-0.6\right)}^{2}-25t+20\\ 1.8\times 10^{3}{\left(t-0.8\right)}^{3}+555{\left(t-0.8\right)}^{2}-5.6t-0.42 \end{array}\right\}. \end{array}$$

**Figure 5:**
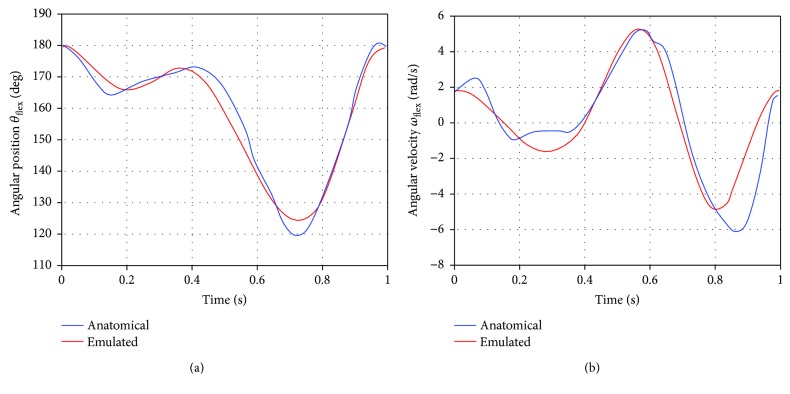
Anatomical (natural [28]) and emulated ((14) and (15)) knee movement: (a) angular position and (b) angular velocity.

The knee motion during a gait cycle was emulated using the previous equations, and the results were compared with the natural knee movement reported in [28]. The results of this comparison are shown in Figure 5, where it can be observed that the proposed fitting curves, (14) and (15), reproduce with an acceptable accuracy the flexion-extension movement of the anatomical knee.

**Figure 6:**
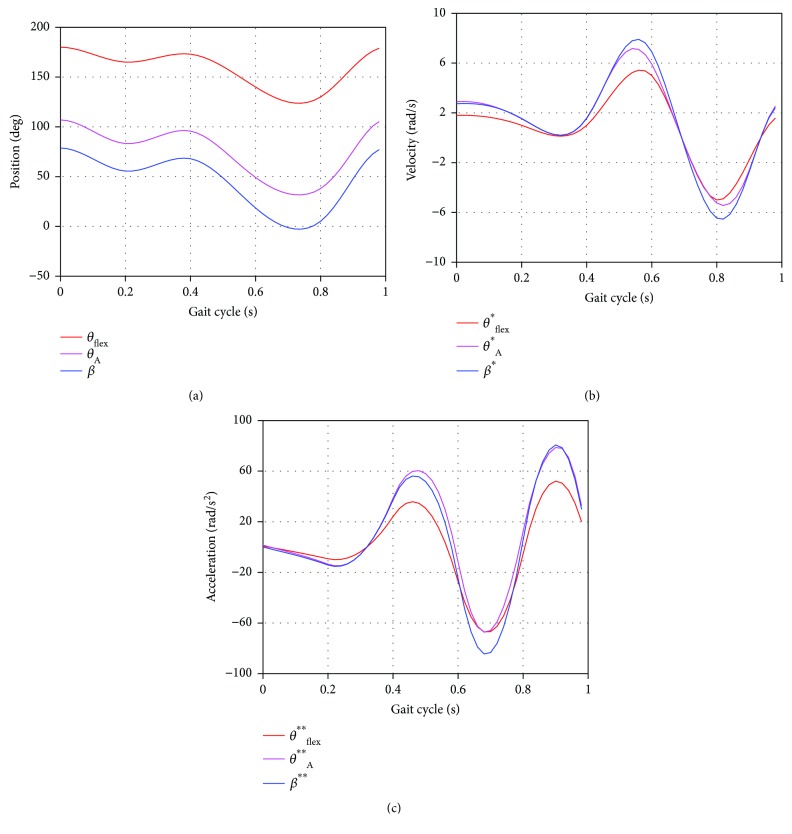
Knee mechanism motion curves for an entire gait cycle (GC): (a) angular position, (b) angular velocity, and (c) angular acceleration.

Since the active joint of the proposed prosthesis mechanism has been defined at node A, it is necessary to determine the input movements *θ*_A_(*t*) and *ω*_A_(*t*) needed to satisfy the knee movements *θ*_flex_(*t*) and *ω*_flex_(*t*). This can be done using (6), (7), (9), (12), (13), (14), and (15). The results are shown in Figure 6 and correspond to a complete gait cycle. From these results, it can be said that the proposed mechanism is able to reproduce the knee flexion-extension motion.

#### 3.4.2. Centrode

**Figure 7:**
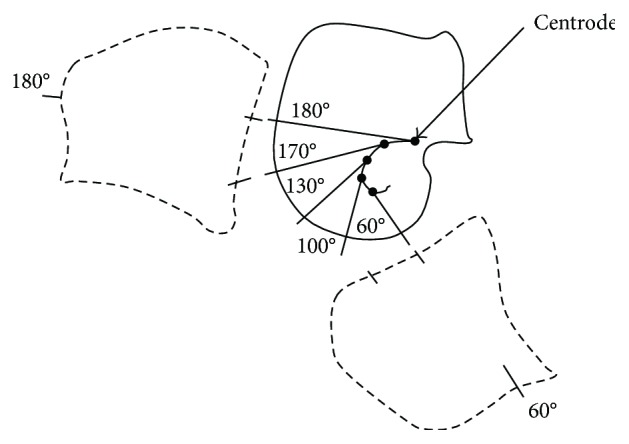
Path line of the ICR during the human knee flexion and extension motion.

The prosthesis centrode, which is defined as the path line traced by the instantaneous centre of rotation (ICR) of the knee mechanism, is fundamental for the stability of the patient. According to [39], the location of the ICR must be in such a manner that an above-knee amputee would have the entire control of the prosthetic device. This is inferred in the assertion that the biomimetized trajectory of the centrode must emulate the path line generated by the ICR of a natural knee. The flexion-extension movement of a human knee is the result of three combined motions [39, 40]: (1) rotation from 180° to 170°, (2) oscillation from 170° to 160°, and (3) translation from 160° to 60°, as shown in Figure 7. From this figure, it can be observed that the ICR of a natural human knee has a smooth path, moving forward and downward as the flexion-extension angle changes.

**Figure 8:**
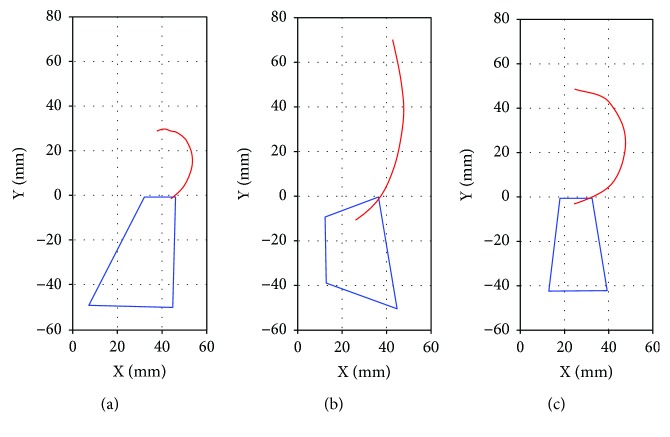
ICR path line for an entire gait cycle (GC): (a) proposed mechanism, (b) Proteval Acphapend prosthesis, and (c) Otto Bock 3R46 prosthesis.

Figure 8(a) shows the centrode of the synthesized mechanism, while Figures 8(b) and 8(c) present the centrode of two commercial prostheses [41]. From these results, it is observed that even though the three prostheses do not reproduce accurately the natural knee centrode (Figure 7), their ICR trajectory shapes are very similar among them. Thus, the proposed knee mechanism is considered feasible in terms of its capability to replicate the human centrode shape.

#### 3.4.3. Leg Shortening

**Figure 9:**
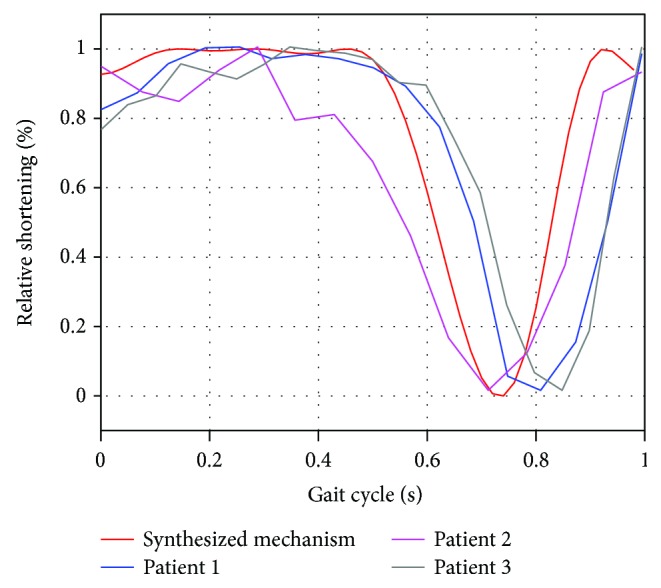
Leg-shortening ability of the synthesized mechanism versus natural knee.

To evaluate the ability of the new proposed polycentric mechanism to replicate the natural leg-shortening effect, a kinematic simulation of the mechanism was carried out using as reference the distance between the middle points of links *L*_2_ and *L*_4_. The variation of this distance along a complete gait cycle corresponds to the leg-shortening effect. The results are shown in Figure 9, which are compared with the observations reported in [16]. From these results, it is observed that the proposed knee mechanism is able to mimic the natural leg-shortening effect, having the smallest length of the leg at 70–80% of the gait cycle, during the swing phase.

### 3.5. Conceptual Design and Modeling

**Figure 10:**
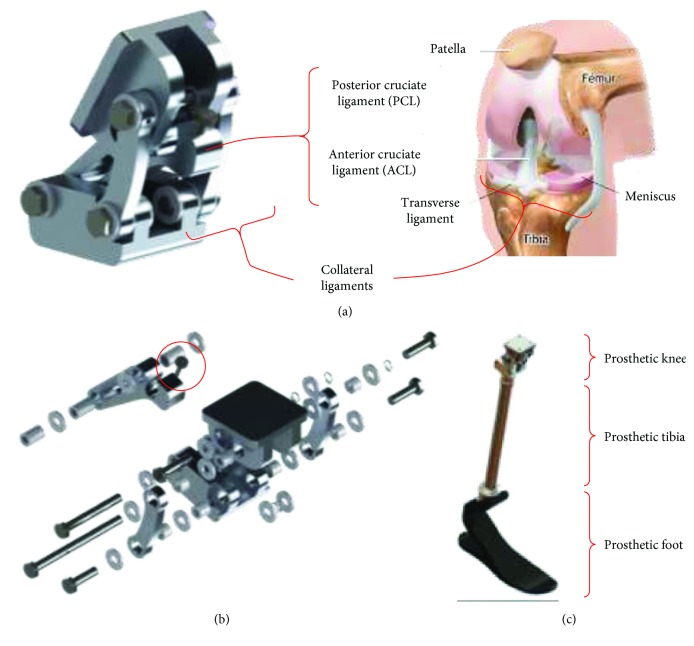
Design of the knee prosthesis: (a) prosthetic knee versus natural knee, (b) exploded view, and (c) integrated transfemoral prosthesis.

Based on the previous results, a 3D model of the knee prosthesis was created in SolidWorks®. The design was inspired by the natural biomimetization of a human knee, comprising two input links that resemble the collateral ligaments and an output link that mimics the cruciate ligaments, as shown in Figure 10(a). Fasteners, pins, links, bushings, and frictional washers were included. The material considered for the links is Al 6061-T6, which has good properties in terms of the strength-to-weight ratio. An exploded view of the polycentric knee prosthesis is shown in Figure 10(b), which has an approximated weight of 359 g. The proposed integrated transfemoral prosthesis includes a 240 g aluminum tibia 2R49 from Otto Bock® and a 345 g carbon fiber prosthetic foot Trias 1C30 from Otto Bock, as shown in Figure 10(c). The Trias 1C30 prosthetic foot corresponds to a dynamic response model recommended for polycentric knee prostheses.

### 3.6. Dynamic Analysis

Since one of the aims of the new prosthesis is to enhance the performance of passive knee prostheses by providing additional power to the patient, a dynamic analysis of the knee mechanism was carried out to determine the input torque *T*_A_ required to move the prosthetic members along a gait cycle on level ground with a cadence of 1 step/s. This additional power is only a fraction of the power needed to fully drive the device and patient. The locking or braking torques associated with other activities, such as climbing stairs, are provided by a physical brake, similar to passive prostheses.

**Figure 11:**
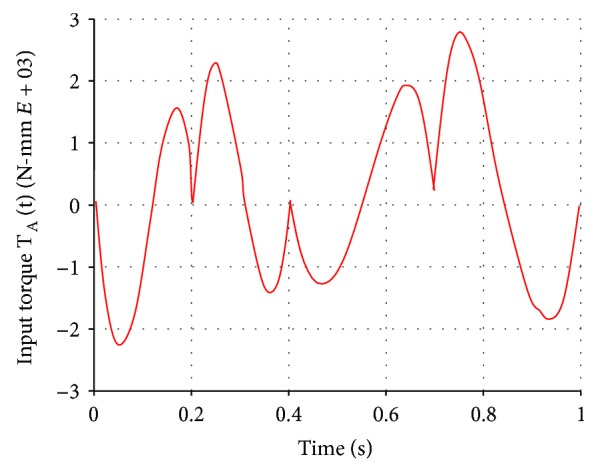
Input torque required for a walking cadence of 1 step/s on level ground.

From the CAD models, the physical variables such as mass, weight, and moments of inertia were obtained. The dynamic analysis was made in the software SAM Mechanism Designer® and SolidWorks. The results are shown in Figure 11, where it can be observed that the maximum value of the input torque during a gait cycle is 2.8 N·m. From these results, a design torque of 3.5 N·m was established considering a total knee prosthesis efficiency of 80%, which includes the efficiencies of the electrical motor, the mechanical drive, and friction in pins [42, 43].

## 4. Control of the Prosthesis

**Figure 12:**
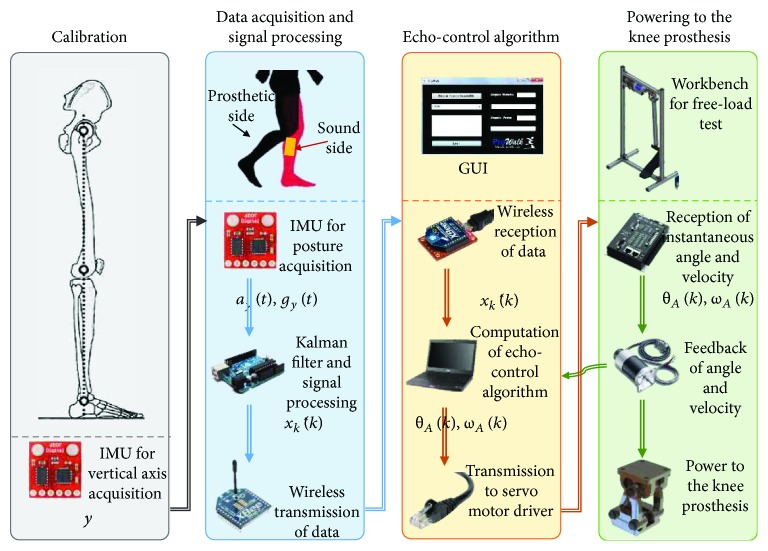
Proposed architecture for the echo-control strategy: (a) calibration, (b) data acquisition and signal processing, (c) echo-control algorithm, and (d) power to the knee prosthesis.

Since physiological human motion, in particular human gait, requires a precise interjoint coordination, an echo-control scheme is proposed to control the knee prosthesis. In this way, the movement required in the active knee prosthesis can be inferred from the signal of the nonimpaired leg (sound side) by means of the echo-control strategy. A modified (mirrored) knee trajectory from the sound leg is calculated and played back on the contralateral side (prosthetic knee) during human gait. The proposed echo-control architecture comprises of four main stages as shown in Figure 12: calibration, data acquisition and signal processing, echo-control algorithm, and powering to the knee prosthesis. Finally, the discrete kinematic parameters *θ*_A_(*k*) and *ω*_A_(*k*) are sent to the last stage to convert them into electric pulses and provide the input power to the knee prosthesis by means of the mastering (conversion from pulses to angle displacement). The current discrete location and velocity of the knee active joint are measured and fed back to the system by means of an encoder located at node A of the knee mechanism.

### 4.1. Calibration

At the calibration stage, the vertical axis *y* of the global coordinate system is set before the first data acquisition. The use of inertial measurement units (IMUs) is proposed for the echo-control system. The IMUs are wearable, portable, lightweight and safe, and accessible for home use, that is, low cost and easy to operate [38]. IMUs measure the acceleration and angular rate in their own three-dimensional local coordinate frame. There is a wide range of IMU calibration methods, including calibration postures and/or calibration movements. The proposed calibration protocol considers the subject standing vertically with straight legs (zero or neutral position) for a few seconds to determine the local coordinates of the segment's longitudinal axis. The IMU is calibrated before the experimental trials in order to determine the initial data for the relative position and alignment. A major issue is the noise produced by the accelerometer when measuring the gravitational acceleration during the back and forth movements of the sound leg. On the other hand, the problem with the gyroscope is that it drifts over the time. Another interference of the sensor signals is the vibration due to the ground contact at the beginning of the stance phase. Thus, it can be said that the gyroscope can be trusted in a short term, while the accelerometer can be trusted in a long term, as presented in [38].

### 4.2. Data Acquisition and Signal Processing

The tilt angle of the tibia $\hat{x}\left(t\right)$ is measured and filtered at the data acquisition and signal processing stage to get ${\hat{x}}_{k}\left(k\right)$. This process involves the measurement and analysis of a group of variables of the system to be controlled. This set of variables is the *state vector* and the *observer*, which are correlated in the equations that describe the physical phenomenon of the dynamic system under study. In the case of the knee prosthesis, the variables to be analyzed are the acceleration and velocity in the vertical direction *a*_y_(*t*) and *g*_y_(*t*), respectively. The aforementioned variables are measured and acquired by means of an IMU. It is required that the average value of the estimated state ${\hat{x}}_{k}\left(k\right)$ must be equal to the average value of the true state $\hat{x}\left(t\right)$. It is also necessary that an estimator is able to provide the smallest possible variation of ${\hat{x}}_{k}\left(k\right)$. A solution to these two requirements is the use of the discrete Kalman filter, which has been proven to be accurate, simple, and robust [33]. Moreover, the discrete Kalman filter complies with the recursion characteristic of a closed-loop control.

For calibration and data acquisition purposes, the following assumptions were made: the IMU is attached in such a way that one of its local coordinate axes is aligned with the knee joint axis; the body of the patient does not have any acceleration, that is, the walking speed remains constant along the gait cycle; the process to be measured can be described by a linear system; the process noise has a Gaussian distribution with an average value of *w* = 0; the average value of the measurement noise *n* is neglected; and no correlation exists between *w* and *n*. The state ${\hat{x}}_{0}$ is initialized as the best initial estimation of position and velocity (${\hat{x}}_{k}\left(0\right)$), at the calibration protocol with a straight standing posture. The error covariance matrix **G**_0_ is initialized as the uncertainty in the initial estimate. As mentioned in [44], these tuning parameters are defined after an optimization procedure.

To start the filtering procedure, the initial estimation of the state vector is zeroed, whereas the initial estimate of the error covariance matrix **G** is set equal to the identity matrix. The last task of the data acquisition and transmission module is to wirelessly send ${\hat{x}}_{k}\left(k\right)$ to the next stage.

### 4.3. Echo-Control Algorithm

At the echo-control algorithm stage, the variable ${\hat{x}}_{k}\left(k\right)$ is received and processed to obtain the kinematic parameters *θ*_A_(*k*) and *ω*_A_(*k*) to control the knee prosthesis. The data reception program uses parallel programming, where the main thread is responsible for receiving information and making operational adjustments to calculate the kinematic parameters from the resulting filtered angle and the current state of the prosthesis (stance or walk). These kinematic parameters are computed based on the complementary (mirror) knee flexion-extension movement of the sound side leg. The second thread is committed to the GUI in order to display the resulting angle of the Kalman filter and the state of the prosthesis (stance or swing phase). The third thread is dedicated to sending the kinematic data to the motor controller via Ethernet.

### 4.4. Powering to the Knee Prosthesis

At the final stage, the kinematic parameters obtained from the echo-control algorithm *θ*_A_(*k*) and *ω*_A_(*k*) are received and transformed into a set of pulses to control and power the motor of the active joint A. The recursion of the control scheme is carried out by feedbacking the real state *θ*_A_(*k*) and *ω*_A_(*k*) to the echo-control algorithm. The real state is measured by means of encoders located at the active joint A.

## 5. Implementation

### 5.1. Mechanical Implementation

**Figure 13:**
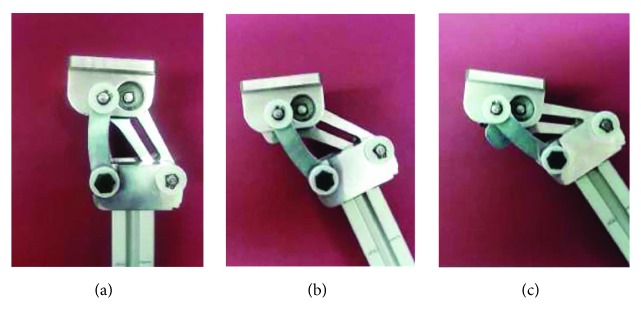
Physical prototype of the knee prosthesis at: (a) stance phase, (b) intermediate phase between support and swing, and (c) maximum flexion phase.

Figure 13 shows the physical prototype of the knee prosthesis at three different positions. All links of the knee mechanism were made of aluminum alloy 6061-T6, whereas the pins of the mechanism were made of low carbon steel, and the bearings and friction washers are standard parts. The amplitude of the flexion-extension knee movement can be adjusted manually by means of a precision screw located at the coupler link, allowing amplitude values from 180° to 165° (hyperextension is avoided).

The total weight of the transfemoral prosthesis is estimated as 3.1 kg, which includes the pyramidal connectors and receivers for the socket and tibia (108 g), the prosthetic foot (345 g), the tibia prosthesis (240 g), the polycentric knee mechanism (359 g), a motor (720 g), a battery (620 g), and fasteners. This total weight is within the normal and acceptable range for transfemoral prostheses and less than a comparable natural limb segment [45]. Moreover, the proposed polycentric knee mechanism is lighter than a similar commercial knee prosthesis, for example, 3R36 Otto Bock prosthesis (445 g), and a similar knee prosthesis from the literature [30].

### 5.2. Control Implementation

A digital IMU with 6 DOF that incorporates the accelerometer ADXL345® and the gyroscope ITG-3200® was used. The IMU was located laterally at the sound side leg in order to measure in real time the motion of the unimpaired leg. The sensors are communicated through I2C using INT0 (gyro) and INT1 (accelerometer) as output markers. In the current algorithm, only the pitch axis is considered for the acquisition of *g*_y_(*t*) and *a*_y_(*t*), required to compute the tilt angle of the tibia. The main computational element of the embedded system is a 16 MHz ATmega328® microcontroller with 32 kB flash memory and 2 kB SRAM. The control and data acquisition is realized via the microcontroller, and the resulting angle of the Kalman filter (tilt angle of the tibia) is sent wirelessly by an XBee® module transmitter. In addition, these modules have lower power consumption and a longer range than Bluetooth transmission. An XBee antenna receives the data to be used in the application program developed in the programming language C#.

The powered prosthesis contains an embedded microcontroller that allows either tethered or untethered operation. The control of the actuator system comprises of the driver board DMC-2143 and the servomotor BLM-N23-50-1000-B, both of Galil®. The motor is controlled by a set of pulses from the operational adaption algorithm and the mastering of them. The knee joint driver includes a planetary gearbox ALPHA LP^+^ [43] with a ratio of *i* = 10. The power drive (motor and gearbox assembly) is capable of an intermittent output torque of 3.7 N·m, for level ground walking with a cadence of 1 step/s.

## 6. Experimental Test

**Figure 14:**
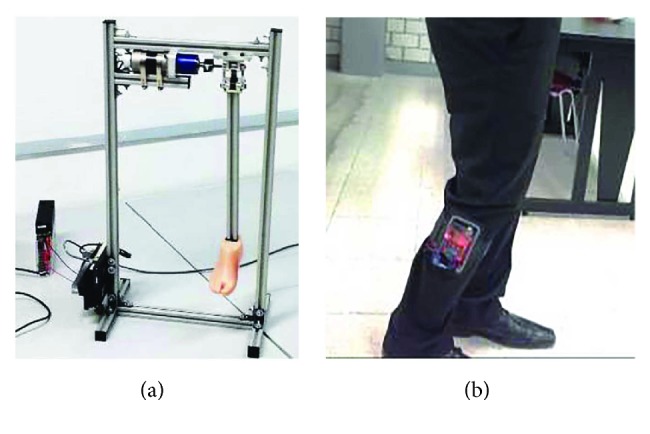
Final prototype of the transfemoral knee prosthesis: (a) test rig and (b) instrumentation of the echo-control technique on the sound side member.

The final knee prosthesis prototype was assembled on a test rig to evaluate its biomechanical performance, see Figure 14(a). Experimental tests were then carried out to confirm that the constructed prosthesis performs as designed. A nonimpaired subject (male, 21 years old, weight 74 kg, and height 182 cm) was equipped with the IMU and asked to walk on a horizontal ground with a cadence of 1 step/s, as shown in Figure 14(b). All the instrumentation of the sound side was located on the top of the gastrocnemius muscle. The experimental tests consisted of the observation and measuring of the gait cycle and the synchronized coordination between the sound side and the knee prosthesis, verifying the angles and state (stance-swing) reported at the GUI. A video was recorded during the tests.

## 7. Results and Discussion

### 7.1. Flexion-Extension Biomimetization

**Figure 15:**
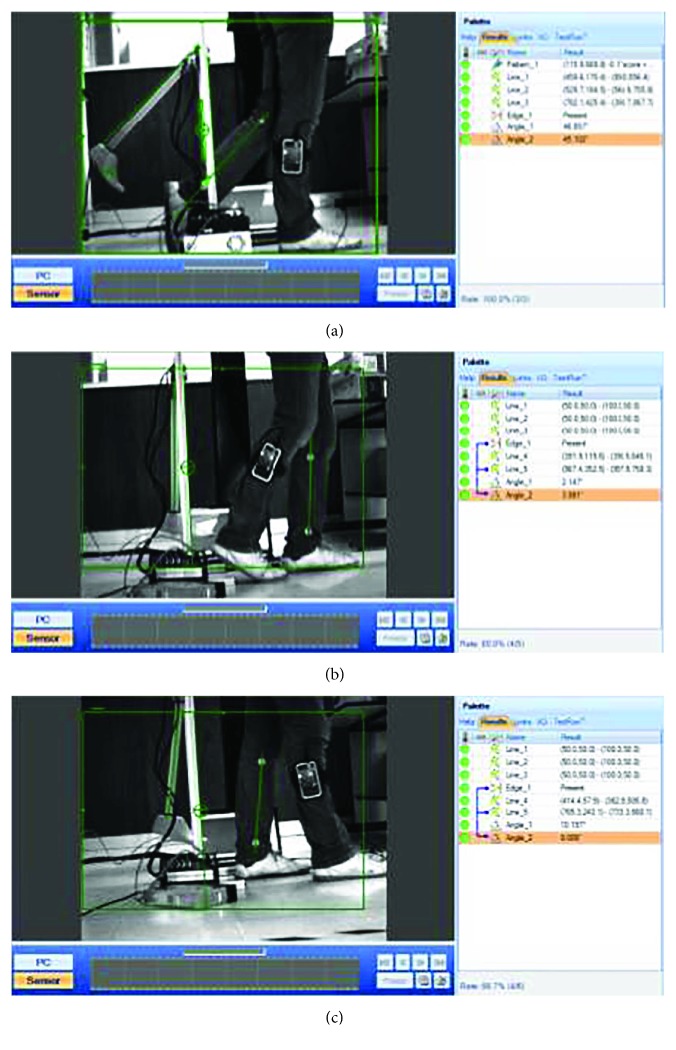
Implementation and evaluation of the knee prosthesis: (a) swing phase, (b) stance phase, and (c) double stance.

The tibia tilt angle $\hat{x}\left(t\right)$ relative to the vertical axis was evaluated and analyzed by means of a computer vision system able to detect in real-time two markers along the axis of the patient's tibia and two markers along the axis of the prosthetic tibia, on the sagittal plane. In the patient, one marker was placed at the top of the ankle and the other was placed under the lateral condyle of the tibia.  Figure 15 shows some of the results obtained from the biomimetical analysis of the tilt angle and the knee flexion-extension movement. These results show that there exists a minimal difference between the tilt angle of the biomimetic-controlled knee prosthesis and the tilt angle of the natural lower limb; this difference is defined as the biomimetic error. The average biomimetic error value was about 2°, which is considered satisfactory based on the natural walk deviation that an anatomical knee provides [46]. Since the tilt angle of the tibia $\hat{x}\left(t\right)$ and the knee flexion-extension angle *θ*_flex_ are supplementary, this biomimetic error also corresponds to the error of the knee flexion-extension movement.

From the experimental results, it can be said that the synchrony prognosis of the flexion-extension movements of the knee prosthesis during human gait was effective and correct. The proposed polycentric knee prosthesis mimics the human movement by echoing the signals acquired from the unimpaired leg. As described in [38], sources of errors include the misalignment of the IMU as a result of the muscle and skin relative motions and the misalignment of the IMU axis with the tibial axis during the calibration procedure. An additional source of error is the noise produced by the oscillation of the IMU signal during the heel contact (initial contact) stage, that is, when the foot impacts the ground. However, the tilt angle required to apply the echo-control strategy was filtered by a Kalman filter, which proved to be an effective method to generate satisfactory results to mimic the human gait.

### 7.2. Centrode Performance

**Figure 16:**
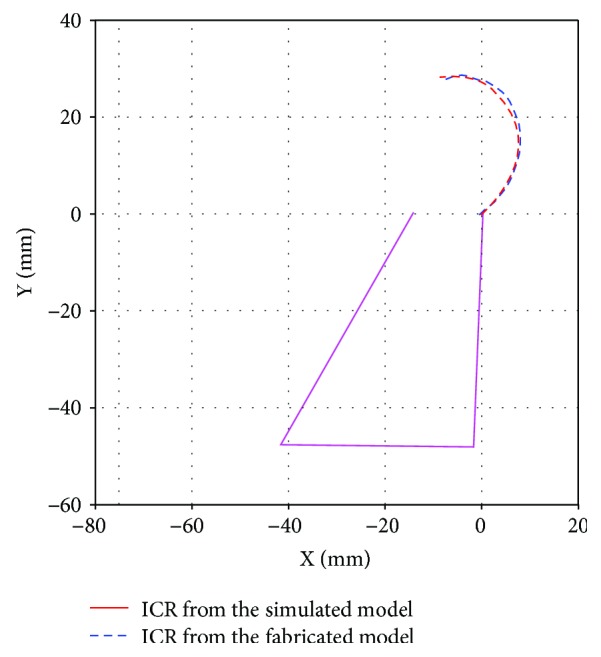
ICR trajectories of the simulation model and the fabricated device.

An image processing analysis was carried out in order to determine the ICR trajectory of the fabricated prosthesis and to compare it with the simulated ICR trajectory. In this analysis, the link *L*_4_ was defined as the fixed reference. The results are shown in Figure 16, where it can be observed that the centrode shape of the prosthesis prototype is very close to the simulated trajectory. The differences between these two trajectories can be attributed to fabrication inaccuracies, as well as the inherent errors of the image processing analysis.

### 7.3. Leg Shortening

To evaluate the leg-shortening performance of the prosthetic prototype, an image processing analysis was also carried out. In this analysis, the link *L*_4_ was also used as the fixed reference, and a vector from the middle point of *L*_4_ to the middle point of *L*_2_ was defined. The results showed that the maximum length during the flexion-extension movement of the prosthesis prototype occurred at approximately 70% of the gait cycle, which is in agreement with the simulation and the natural knee movement. Thus, it can be said that the prosthetic prototype is able to reproduce the leg-shortening effect.

### 7.4. Control

The advantages of implementing wearable sensors were identified, which includes the possibility to move anywhere without controlled environments, the portability, and the promotion of patients' autonomy. The practicality of the echo-monitoring strategy by obtaining the necessary signals from the sound side and inputting them to the algorithm of the echo-control strategy was also confirmed. Moreover, the proposed echo-control approach improves some of the weaknesses, such as the complexity and instability, of prior strategies reported in the literature. However, some limitations of the echo-control strategy were also identified, in particular, at the beginning and end of activities when the legs do not have to echo one another. In such cases, a phase shift is not necessary, and the movement of the sound side could be mapped directly into the impaired limb. Such an approach supposes the symmetry of motion between the two limbs, which may be also inappropriate in cases where bilateral assistance is required or where the required gait pattern is inherently asymmetric, agreeing with [47]. It is also remarkable that any nondesired movements will also be mapped. Additionally, the echo-control strategy may induce problems when an odd number of steps must be performed.

Finally, it can be said that the proposed biomimetic-controlled transfemoral knee prosthesis based on a polycentric mechanism and an echo-control scheme is able to reproduce the biomechanical performance of a natural knee. However, more work is needed in order to enhance the prosthesis design in terms of topology optimization, portability, aesthetics, and its evaluation on a real amputee.

## 8. Conclusions

This paper has presented the design and analysis of an active polycentric transfemoral knee prosthesis able to biomimic the human gait. The proposed knee prosthesis is based on a four-bar mechanism obtained from a synthesis process to reproduce the natural human knee movement. An echo-control strategy has been proposed for the activation of the knee prosthesis. The experimental results with a nonimpaired subject have demonstrated that the proposed prosthesis is able to biomimic the biomechanics of a natural knee during gait and to provide extra power to the transfemoral amputee during walking. The results have also shown that the minimization of “autonomous intelligence” in an actuated prosthesis, in combination with the close observation of the user, allowed the incorporation of the human's superior control in a cooperative and intuitive way. Finally, it can be concluded that the echo-control strategy is an effective control approach to biomimic human movements because of its simplicity, low cost, portability, and accuracy.

Future work comprises of the topological optimization of the knee mechanism in order to reduce its weight. The portability of the proposed prosthesis is also part of the future work in order to carry out tests on a real amputee. In addition, the application of a similar control scheme for knee prostheses with dissipative devices, which offer considerable advantages in terms of weight and range, is also considered as future work.

## Figures and Tables

**Table 1:** Desired locations of the coupler link joints.

Desired *i*th configuration	A_*i*_ (mm)	R_*i*_ (mm)
1	(12.0, −76.0)	(−27.5, −72.0)
2	(0.0, −73.5)	(−38.5, −63)
3	(−31.5, −37.7)	(−49, −2.8)

**Table 2:** Result of the synthesized polycentric knee mechanism.

Link	Dimension (mm)
Input link: *L*_1_	48.28
Coupler link: *L*_2_	40.00
Output link: *L*_3_	55.43
Fixed link: *L*_4_	14.26
